# Benefits of applying molecular barcoding systems are not uniform across different genomic applications

**DOI:** 10.1186/s12967-023-04160-0

**Published:** 2023-05-05

**Authors:** Jonathan Bieler, Slawomir Kubik, Morgane Macheret, Christian Pozzorini, Adrian Willig, Zhenyu Xu

**Affiliations:** Data Science Department, SOPHiA GENETICS, Rue du Centre 172, CH-1025 Saint Sulpice, Switzerland

**Keywords:** Genomics, Variant calling, Molecular barcode, Unique molecular identifier, UMI

## Abstract

**Background:**

Despite the wide variety of Next Generation Sequencing (NGS)-based methods, it remains challenging to detect mutations present at very low frequencies. This problem is particularly relevant in oncology, where the limiting amount of input material, and its low quality, often limit the performance of the assays. Unique Molecular Identifiers (UMIs) are a molecular barcoding system often coupled with computational methods of noise suppression to improve the reliability of detection of rare variants. Although widely adopted, UMI inclusion imposes additional technical complexity and sequencing cost. Currently, there are no guidelines on UMI usage nor a comprehensive evaluation of their advantage across different applications.

**Methods:**

We used DNA sequencing data generated by molecular barcoding and hybridization-based enrichment, from various types and quantities of input material (fresh frozen, formaldehyde-treated and cell-free DNA), to evaluate the performance of variant calling in different clinically relevant contexts.

**Results:**

Noise suppression achieved by read grouping based on fragment mapping positions ensures reliable variant calling for many experimental designs even without exogenous UMIs. Exogenous barcodes significantly improve performance only when mapping position collisions occur, which is common in cell-free DNA.

**Conclusions:**

We demonstrate that UMI usage is not universally beneficial across experimental designs and that it is worthwhile to critically consider the comparative advantage of UMI usage for a given NGS application prior to experimental design.

**Supplementary Information:**

The online version contains supplementary material available at 10.1186/s12967-023-04160-0.

## Background

Next-generation sequencing (NGS)-based methods are undisputed as the premier tools for the detection of genomic alterations in research and diagnostics. Nevertheless, fully leveraging their potential requires accounting for technological and analytical factors that limit the power of these approaches. Notably, identification of mutations with low frequency in the input sample presents a significant challenge. Reliable detection of such mutations is particularly crucial for oncology applications, as the nucleic acid extracted from tumor tissue samples is derived from a heterogeneous cell population with diverse mutational profiles across subclones. Similarly, high-throughput sequencing of circulating tumor DNA (ctDNA) promises to enable personalized cancer therapy [1]; however, limited quantities of cell-free DNA (cfDNA) present in blood as well as high levels of noise relative to mutation frequency currently limit the analytical performance for this application. Indeed, the concentration of cfDNA in plasma ranges from 1 to 100 ng/mL [2], and the frequency of mutations is often below 1% [3]. Presenting even more of a challenge, minimal (or “measurable”) residual disease (MRD) monitoring, either from ctDNA or white blood cell DNA, requires confident variant calling at fractions of 0.1% or lower [4, 5]. Detection of such rare events calls for careful considerations on the type and quantity of the input material, conversion of input molecules, the sequencing depth and noise suppression methods. Currently, the above aspects are not standardized across clinical laboratories as no clear and consistent guidelines for MRD detection by NGS exist. It is crucial to understand the experimental and analytical limits to NGS experimental designs to leverage limited material most effectively for the identification of low-frequency mutations.

**Fig. 1:**
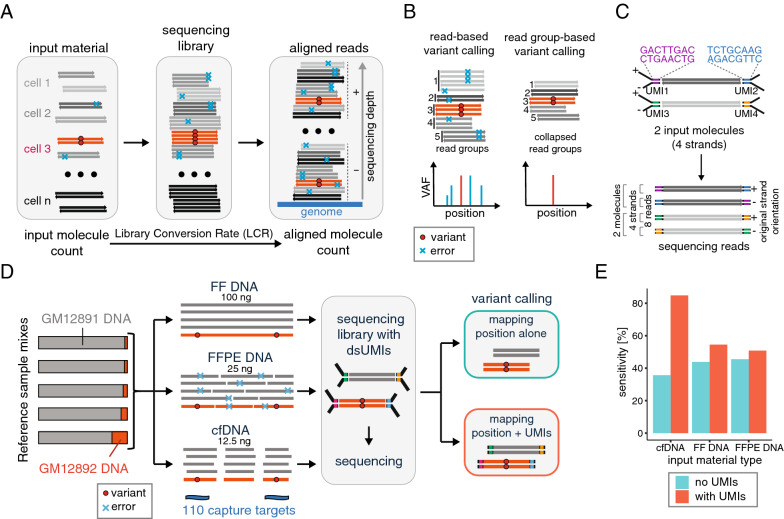
UMI usage does not provide consistent improvement across experimental setups. **A** Schematic representation of signal and noise generation from the input DNA in a typical NGS experiment. **B** Schematic representation of the principle of noise suppression by read grouping prior to variant calling; calls recognized as noise (i.e. not found consistently across reads derived from both strands within a read group) are ignored during read collapsing. **C** Schematic depiction of a double-stranded UMI system (top) and its use to identify 2 input molecules from 8 sequencing reads (bottom). **D** Schematic representation of the experimental setup used to investigate variant calling performance in different types of input material; 3 types of input (FF, FFPE and cfDNA) were generated from mixtures of the reference samples GM12891 and GM12892 at various ratios (see the “Methods” section); the mixes were used to generate libraries that were subsequently enriched using hybridization capture with a panel targeting 110 variants and then sequenced; after sequencing and read alignment, variant calling was performed using read mapping positions alone or using mapping positions and UMI information. **E** Sensitivity of duplex consensus-based variant calling (using a threshold of at least 2 duplexes to call) for 110 known variants present at VAF of 0.2% in three types of input material (12.5 ng cfDNA, 100 ng FF DNA, 25 ng FFPE DNA) with read grouping either using mapping positions alone or using mapping positions plus UMI information

A robust NGS assay is required to provide exact qualitative (i.e. correct sequence) and quantitative (i.e. number of molecules) sequence information for input molecules. However, the ground truth sequences undergo multiple rounds of processing before the output result. Each of the processing steps has the potential to introduce noise in the form of sequence errors or quantitative bias. Errors can result from damage to the template molecule, issues with input preparation (*e.g.* end repair and A-tailing), ligation of adapters, amplification and enrichment steps and those introduced during the sequencing process itself [6] (Fig. 1A). Low frequency variant calling is hampered by the presence of sequence errors giving rise to signal comparable to, or higher than, the one of true mutations.

Library conversion rate (LCR) reflects what fraction of the input molecules are represented in the sequencing data. Importantly, a single input molecule may be represented by many duplicate reads with distinct sequence errors. Therefore, some portion of sequencing noise can be suppressed by grouping reads according to the input molecule from which they originate and determining the correct base call at each position within a group from the base frequency and read quality in a de-duplication process (Fig. 1B). In Illumina sequencing, each of the strands of an input DNA molecule may yield a population of library molecules, and ultimately reads, that can be tracked to either the original forward or reverse strands. As the DNA strands get separated during the first PCR cycle, errors—even those occurring early in the amplification process—will be present in the reads derived from only one of the strands. This information can then be used to increase confidence for calls consistent across both strands [7, 8]. A common mechanism for assigning reads to groups is the addition of molecular barcodes to the input molecules prior to amplification (Fig. 1C). Such barcodes, referred to as unique molecular identifiers (UMIs), consist of a string of either random, semi-random, or predefined nucleotides [9]. UMIs are generally recognized as an efficient tool for noise suppression by facilitating read grouping and collapsing prior to variant calling, ultimately improving the efficiency of mutation detection [9–11]. Due to this advantage, UMI implementation has become a standard procedure across many NGS applications. However, the usage of UMIs increases experimental complexity and cost and it is not clear if it provides significant advantage across various experimental setups. For instance, hybridization capture-based methods, unlike amplicon-based assays, enable read grouping without the addition of exogenous UMIs. Owing to random fragmentation of DNA, read mapping positions can be used as endogenous molecule identifiers that facilitate the assignment of read groups and allow for mapping position-based variant calling. Importantly, the value of exogenous UMI-based read grouping as compared to mapping position-based grouping has not been rigorously tested for clinical applications. To address this gap, we undertook systematic analysis of sequencing performance in the presence and absence of exogenous UMIs for various clinically relevant experimental contexts. We observed that the advantage of using UMIs in hybridization-based methods depends on the experimental setup, namely the amount of sequenced molecules and their fragmentation pattern.

## Methods

### Preparation of reference cfDNA samples

Nucleosomal DNA was generated from two different human cell lines (GM12891 and GM12892, Coriell Institute) with the EZ Nucleosomal DNA Prep (Zymo Research, Cat. No. D5220) using the Atlantis dsDNase treatment according to the manufacturer’s instructions with minor modifications, as follows. After collection, cells were stored at −80 °C. For each cell line, a pellet of 10^6^ cells was thawed on ice, resuspended in 1 ml ice-cold lysis buffer (10 mM Tris–HCl pH 7.5, 10 mM NaCl, 3 mM MgCl2, 0.5% NP-40, 0.5 mM Spermidine) and incubated for 5 min on ice. The resulting nuclei were washed once with 200 μl ice-cold Atlantis digestion buffer, resuspended in 100 μl Atlantis digestion buffer with 0.25 U Atlantis dsDNAse and incubated for 1 h at 42 °C. To stop the digestion, 18 μl of Stop solution (75 mM EDTA, 1.5% SDS, 0.7 M NaCl) was added. Samples were then treated with RNaseA (0.3 mg/μl) for 15 min at 42 °C, then with proteinase K (1 mg/ml) for 30 min at 37 °C. Following enzymatic treatments, DNA was column purified with the Zymo Genomic DNA Clean & Concentrator kit (Zymo Research, Cat. No. D4065). To remove excessively large and small DNA fragments, samples were subjected to a dual size selection using AMPure XP Beads (Beckman Coulter) with bead:DNA ratios of 0.7 × and 1.5x. After fluorometric quantification, the DNA of cell lines were combined, generating DNA mixes with variants at the desired variant fractions.

### Preparation of reference FFPE samples

To mimic clinically derived formalin-fixed paraffin-embedded (FFPE) material using reference samples, DNA was generated from two different human cell lines (GM12891 and GM12892, Coriell Institute) and pellets subject to formaldehyde treatment. Briefly, pellets containing 30–50 million cells were flash-frozen on dry-ice and carefully submerged in 15 ml of neutral buffered formalin or zinc formalin without disturbing the pellet. Cells were incubated for 72 h at 37 °C. Formalin was removed and replaced with 20 ml of 70% EtOH without resuspending the pellet. The cells were kept at 4 °C until further processing.

The pellet was dehydrated with the Tissue-Tek VIP6 robot (Sakura) using the nerve program. The cassette with the pellet was subsequently embedded in paraffin. Scrolls were created by sectioning the paraffin block with the microtome (thickness of 15 µm).

### Sample mixing for variant calling performance

To investigate the performance of variant calling for different types of input material, DNA of reference samples GM12891 and GM12892 (Coriell Institute) were mixed at ratios at which the final VAFs in the mix, for variants heterozygous in GM12892 and absent in GM12891, were 0.05%, 0.1%, 0.2%, 0.5%, 1% or 2%. Such mixes were generated in replicates, using 3 types of input material: fresh frozen DNA (FF DNA, 100 ng), FFPE DNA (25 ng) and cfDNA (12.5 ng). For measurements of collision rates, DNA of GM12891 alone was used as input for library preparation. See Additional file 1: Table S1 for details on all the libraries prepared.

To investigate the extent of variant signal loss occurring due to collisions, and for each type of input DNA, FASTQ files derived from sequencing of DNA mixes with various VAFs were merged with combinations of FASTQ files derived from pure GM12891 sample (e.g. 0.5% mix + 25 ng of GM12891, 0.5% + 25 ng + 5 ng of GM12891, etc. see Fig. 4D). This allowed to obtain conditions at which variant fraction was “diluted” by the background sample in a manner proportional to the total input amount of the background samples used. Even with modest number of pure GM12891 samples the total number of possible combinations can grow very large. Thus the number of combinations was limited by imposing a minimum total input amount difference between two successive combinations (FF: 20 ng, FFPE: 10 ng, cfDNA: 5 ng) and keeping only one combination per total input amount.

### Targeted libraries preparation & capture panel

Targeted libraries were created using capture-based enrichment technology including molecular barcodes. Various types and amounts of DNA, including cfDNA, FFPE DNA and FF DNA, were used for library preparation with KAPA HyperPlus kit (Roche). Briefly, DNA was enzymatically fragmented (for FF DNA only), end-repaired and A-tailed, followed by ligation to custom short Y-shaped adapters with a double-stranded molecular barcode of 4–5 bp. The ligation products were purified with AMPure beads (Beckman Coulter) and then amplified by PCR for 10 to 14 cycles, depending on the amount of input DNA, using Illumina-compatible primers with dual-indices. Amplified libraries were cleaned-up with AMPure beads (Beckman Coulter).

The pools were mixed with human Cot-1 DNA (Life Technologies) and xGen Universal Blockers-TS Mix oligos (Integrated DNA Technologies) and lyophilized. Pellets were resuspended in a hybridization mixture, denatured for 10 min at 95 °C and incubated for 4–16 h at 65 °C in the presence of biotinylated probes (xGen Lockdown, IDT^®^). The probe panel spanned 46 kbp and covered a set of 110 selected loci bearing variants (70 SNVs, 20 deletions, 20 insertions) present in the GM12892 genome and absent in GM12891 [12]. Probe-hybridized library fragments were captured with Dynabeads M270 Streptavidin (Invitrogen), washed, amplified by PCR for 15 cycles and purified using AMPure beads (Beckman Coulter).

### Sequencing

All libraries were sequenced using a NovaSeq sequencer (Illumina) on a S4 lane as specified by the manufacturer. Paired-end sequencing reads of 150 bases were generated (2 × 150 cycles) together with two index reads of 8 bases. Libraries were loaded on the sequencer such that coverage was proportional to the input material aiming to obtain at least 5 reads per molecule on average. This guarantees that most molecules converted into library were sequenced at least once.

### Statistical analysis

#### Data processing

Demultiplexing and molecular barcode trimming was performed on base-call files (BCL) using bcl2fastq2 and the resulting FASTQ files were aligned against the human genome reference hg19 (GRCh37.p5) using BWA [13]. All post-alignment processing and analysis was done using custom tools written in the Julia language [14].

#### UMI processing & molecular counting

UMIs were trimmed during demultiplexing and appended to the read name. The UMI sequences were then used to group fragments sharing the same mapping position, with a tolerance of one mismatch to account for sequencing errors. When ignoring the UMIs in the analysis, the fragments were grouped based on mapping position alone. The molecules were further classified as simplex or duplex depending on the presence of one or both strands in the group. When counting molecules, both simplex and duplex were included.

#### Duplex variant calling & analytical performance

Groups of PCR duplicates were collapsed to consensus sequences prior to pileup, based upon a previously described method [7]. Two alternative approaches were used for read grouping: pairs of reads were grouped into DNA molecules either using their mapping positions alone (labeled in figures as "no UMIs”) or using the mapping position and molecular barcode sequence information together (“with UMIs”). Molecules for which only one of the two strands were sequenced were discarded and at least 3 reads in the group were required to form a consensus. For each remaining molecule a base call consensus was obtained for each strand. At least 70% base call consistency within a strand was required to form a consensus. Finally, a consensus was obtained for both strands of each molecule, producing a single high-confidence sequence for each DNA molecule. Alternative bases were required to be present in both strand consensus sequences to be retained in the final representation.

For a given position, a pileup of the consensus sequences was performed and the number of molecules supporting a given variant was counted. Variant calls were made by applying a threshold to that molecular count.

Sensitivity was computed as the fraction of confirmed variants that were called. Specificity was computed as the ratio of false calls to all theoretically possible false calls across all interrogated positions where no variant is expected. The number of theoretical false calls equals to the number of positions with no expected variant multiplied by 5 (for three possible single nucleotide changes, an indel and a deletion). Since more than 1 false positive is possible per position, this ensures that specificity remains in a range between 0 and 100%. To make the value easier to interpret, specificity was expressed as the number of false positive calls per 1 kb.

The standard receiver operating characteristic (ROC) curve was computed by changing the threshold on the required number of molecules supporting a variant and the area under the curve (AUC) was obtained by numerically integrating the ROC.

#### Model for the probability of sequencing one alternative molecule at a given VAF

Given a number of molecules $$N$$ and a VAF it is possible to evaluate the probability of sequencing at least one molecule carrying the alternative allele ($${N}_{alt}=1)$$. Assuming each molecule is sequenced at least once (which should be the case when one wants to perform noise suppression with UMIs), the *N*_*alt*_ follows a binomal distribution,$${N}_{alt} \sim Binomial(N, VAF)$$, and thus the probability of sequencing at least one molecule is given by:$$1 - Binomial(N, VAF; 0)$$.

#### Model for the relation between input amount and molecular count

Approximately 300 genomic equivalents are found in 1 ng of DNA, so that for a given input amount in nanograms, and a given LCR, the average molecular count is given by: $N= 300 \times Input \times LCR$.

## Results and discussion

### Benefits of applying molecular barcoding are not uniform across NGS-based applications

To assess the impact of exogenous UMIs on the efficiency of rare mutation detection, we prepared FF DNA, FFPE DNA or artificial cfDNA using characterized reference samples (see “Methods” section). Artificial cfDNA was generated using enzymatic cleavage which, unlike sonication-based shearing, yields material with a fragmentation pattern closely resembling clinical samples [12]. As input, we used quantities that are within range typically encountered in clinical settings (100 ng FF DNA, 25 ng FFPE DNA and 12.5 ng cfDNA). Sequencing libraries were prepared using adapters bearing double-stranded UMIs. Regions containing 110 confirmed variants, present at variant allele fraction (VAF) of 0.2%, were subsequently enriched using a hybridization-based approach (Fig. 1D). We compared variant calling results using mapping positions alone or using additional UMI-based information for read grouping. Analysis of variant detection sensitivity revealed that the performance achieved with the UMIs was not significantly different (p > 0.05, Fisher's exact test) from the one obtained using only the fragment mapping positions for FF (54.5% vs 43.9%) and FFPE (50.8% vs 45.5%) samples. In contrast, a significant improvement (p < 10^–7^, Fisher's exact test) in sensitivity was observed for cfDNA when UMI information was considered (84.8% vs 35.7%) (Fig. 1E). These results suggested that the impact on performance is not consistent across different sample types when comparing applying UMI information versus using mapping positions alone for read group assignment. Given this observation, we dissected the differential effects of UMI usage considering the major factors driving efficiency of variant detection and reviewing read group-based variant calling in different scenarios.

### Signal and noise levels depend on input material and experimental design

The benefit of UMI usage relies on suppressing sequencing read noise while maintaining the signal. We sought to investigate the relationship between these two processes from the perspective of different experimental conditions. In an NGS experiment, “signal” refers to the sequence mapped to a given genomic position that correctly reflects the sample genotype. The signal is impacted by errors introduced at multiple workflow stages (“noise”). Reliable variant calling requires (i) efficient signal retrieval (avoiding false-negative and false-positive calls) and (ii) separation of the signal from noise (discriminating true-positive from false-positive calls). The type of available biological specimen determines the input material characteristics and therefore the expected output data features, including noise levels.

**Fig. 2:**
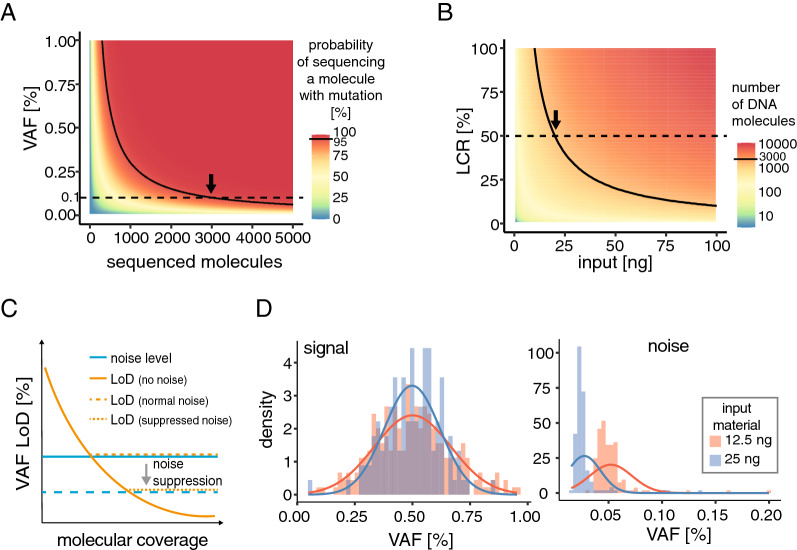
Input material determines signal and noise levels. **A** Binomial probability of mutation-bearing molecule detection (color scale) depending on the number of molecules tested (x axis) and the fraction at which mutation is found in the input (y axis); solid line indicates 95% probability; the arrow indicates number of molecules required for 95% probability of detecting mutation present at VAF of 0.1%. **B** Theoretical number of molecules sequenced depending on the amount of input DNA (x axis) and library conversion rate (y axis); solid line indicates 3000 molecules; arrow indicates input required for 3000 molecules at 50% LCR. **C** Limit of signal detection in NGS data (LoD, orange line), expressed as minimal VAF that can be reliably detected for true variant calls, is limited by the level of generated noise (blue line); LoD can be decreased by noise suppression with analytical approaches. **D** Distribution of VAF measured for true signal (left) or noise (right) using hybridization capture assay, in a sample bearing 110 variants at expected frequency of 0.5%, where 12.5 ng (red) or 25 ng (blue, obtained by merging FASTQ files of 12.5 ng replicates) of cfDNA was used as input

The chance of detecting a given mutation can be described by a binomial distribution depending on how frequently the mutation is present in the input genomes and how many molecules overlapping the position of interest are sequenced (Fig. 2A, Methods). For example, achieving a 95% chance of detecting a mutation present at VAF = 0.1% (crucial for MRD monitoring from cfDNA) requires sequencing at least ~ 3000 molecules overlapping the mutated position, even in the absence of noise. The number of sequenced molecules (molecular count) is determined by (i) the amount of input material (total available molecules), (ii) LCR (fraction of input molecules converted to library) and (iii) the sequencing depth. Approximately 300 genomic equivalents are found in 1 ng of dsDNA. Therefore, to sequence 3000 genome equivalents, a method with LCR of 50% (value reported previously for cfDNA processing [15]) would require at least 20 ng of input DNA (Fig. 2B). Consequently, achieving 95% sensitivity for detection of low frequency mutations may prove impossible when very little input material is available or when the method LCR is low. Given the number of duplexes obtained in our experiment (about 1′000 on average for 100 ng FF DNA, 950 for 25 ng FFPE DNA and 1′900 for 12.5 ng cfDNA), the mean probabilities of detecting at least two variant molecules at 0.2% VAF were 59%, 56% and 89% for FF, FFPE and cfDNA respectively. Notably, our variant calling approach necessitated conversion of both strands for variant call, and the FF condition showed relatively low duplex recovery rate, possibly due to suboptimal library preparation or capture conditions. Good agreement with observed sensitivities (54.5%, 50.8% and 84.8%, respectively, Fig. 1E) confirms nonetheless that the molecular count is a major determinant of sensitivity.

Achieving adequate molecular coverage is required, but not sufficient for reliable variant detection, since transmission of molecule sequence information to sequencing data may not be stoichiometric. Biases relate to the fact that certain molecules (or even individual DNA strands) can be more likely than others to efficiently progress through any step of the protocol [16]. A bias can occur at the step of template preparation, amplification, fragment size selection, enrichment, and/or sequencing and it is driven by molecule-specific factors such as the molecule AT content, among others (Additional file 2: Figure S1A).

Several factors determine the limit of detection (LoD) for the frequency of identifiable alterations [12]. Even if in a simplified theory the LoD can reach arbitrarily low values with sufficient molecular coverage, in practice it is in fact limited by noise in the sequencing reads. Importantly, there are various biochemical or computational solutions to reduce the amount of noise and minimize or correct for bias (Fig. 2C).

Abundant, high-quality material can be obtained from fresh frozen tissue, blood, saliva, or urine samples. However, for solid tumors, biopsy-derived tissue samples are typically FFPE-treated prior to DNA or RNA isolation, often resulting in physically and chemically degraded material, thereby hampering efficient library conversion and inflating errors. Cell-free DNA is typically of good quality but is highly fragmented and available in small quantities. As expected, in the samples presented in Fig. 1E, the false positive rate (for a fixed threshold for a call of at least two duplexes) was 2.3 FPs per kb for FFPE, 0.44 for FF and 0.03 for cfDNA. Therefore, the input material has a significant impact on the signal to noise ratio for downstream sequencing applications.

Part of noise in the measured variant fraction is caused by random sampling, and this becomes elevated when molecular count is low. As a result, the variance of the signal is higher and errors reach higher frequencies. This is clear when comparing signal and noise levels in data generated with varying input amounts of the same sample (Fig. 2D). Since obtaining high amounts of input material is challenging in certain contexts (*e.g.* cfDNA), maintaining high conversion rate of the starting material coupled with efficient methods of signal detection and noise suppression is necessary to maximize information recovery.

### Collisions decrease the sensitivity of mapping position-based variant calling

Assigning reads to groups and employing de-duplication to identify input molecules before variant calling significantly reduces the noise in sequencing data. However, not every NGS application requires such noise suppression. For example, good quality DNA processed with high LCR, such as white blood cell DNA, can yield data with error levels low enough not to benefit from read grouping before variant calling at a desired VAF. On the contrary, variant calling in data generated with FFPE samples, which is characterized by high noise levels and low molecule counts, may greatly benefit from noise suppression via read grouping. Multiple approaches exist for assigning reads to groups and using this information to infer input molecule sequence [7, 10, 11]. For all presented data in this work, duplex-based molecule sequence determination was used to call variants (see “Methods” section).

**Fig. 3:**
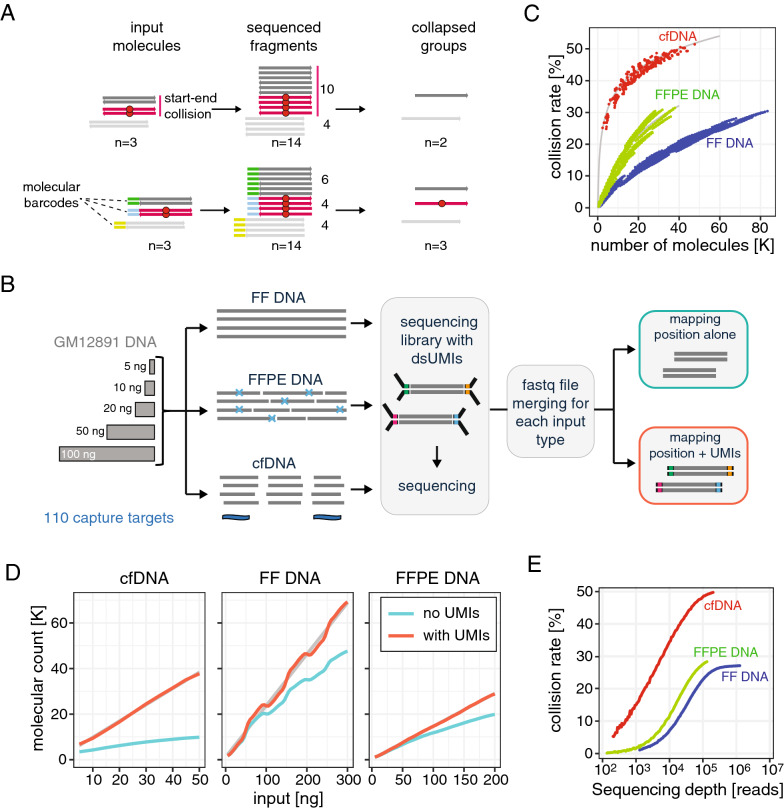
Collisions affect group-based variant calling methods. **A** Schematic showing read group-based variant calling in the presence of collision. In the absence of exogenous barcodes (top), variant information is lost due to colliding reads without the variant outnumbering those with the variant. Exogenous barcodes allow tagging of the original input molecules (bottom) and preserve this information for the sequencing reads, leading to successful detection of the variant. **B** Schematic representation of the experimental setup used to investigate collisions in different types of input material; 3 types of input (FF, FFPE and cfDNA) were generated from the reference sample GM12891; various input amounts were used to generate libraries that were subsequently enriched using hybridization capture with a panel targeting 110 loci and then sequenced; resulting FASTQ files were optionally merged to obtain higher number of total sequenced molecules for each input type; after sequencing read alignment, variant calling was performed using read mapping positions alone or using mapping positions and UMI information. **C** Collision rates plotted for three different types of input DNA (with input amount ranging from 5 to 50 ng of cfDNA, 5–200 ng of FFPE DNA and 5–300 ng of FF DNA) as a function of the number of sequenced molecules (estimated using mapping position and UMIs). **D** For the three types of input DNA, plots showing the number of molecules calculated using mapping positions alone (cyan) or mapping position and UMI information (red), as a function of DNA input amount. **E** Collision rate as a function of sequencing depth for three different types of DNA. Plots in (**C**), (**D**) and (**E**) were generated from FASTQ files obtained by merging all files obtained for a given type of input

Despite read group-based variant calling that relies on mapping positions being a powerful and simple method of noise suppression, it can fail in situations where individual input molecules share the same mapping positions – an event known as “collision”. As a result, if a variant is present in a read group containing fewer duplicates than the colliding group (Fig. 3A, top), the variant information will be ignored upon read collapsing.

Collision rate, defined as the fraction of read groups that display at least one collision, as inferred from sequencing data, depends on the fragmentation pattern of the input material. Therefore, it is expected to be low in methods applying random shearing of DNA (sonication or enzymatic cleavage) and reach 100% for amplicon-based methods where molecule boundaries are predefined by primer positions. Exogenous UMIs can be used to measure and resolve collisions by uniquely tagging the starting molecules (Fig. 3A, bottom). The sensitivity of amplicon-based assays without UMIs may be lower than that of hybridization capture methods, as they cannot rely on molecule distinction by mapping position [12].

To systematically address the impact of collisions on sequencing sensitivity of different samples, we measured collision rates for different types of input material—FF DNA, FFPE DNA and cfDNA—generated from the same reference sample (GM12891) and captured with a hybridization-based approach (Fig. 3B). Mapping positions and UMI information were used to identify individual molecules. Analysis of the collision rate in function of the number of molecules revealed that cfDNA samples are characterized by particularly high collision rates (~ 44% with 20′000 sequenced molecules using 50 ng input) when compared to FF (~ 12% with 20′000 sequenced molecules using 300 ng input) and FFPE-derived (~ 23% with 20′000 sequenced molecules using 200 ng input) DNA (Fig. 3C). This is a direct consequence of cell-free DNA being generated by nucleases, a process guided by nucleosome positioning [17]. Basic units of chromatin are composed of ~ 147 bp of DNA wrapped around each nucleosome core particle and short intervening linkers in between. During chromatin degradation, the linkers are much more accessible to nucleases than DNA in the core, resulting in a restricted set of highly frequent fragment edges [18].

Importantly, higher collision rates in cfDNA than in FF and FFPE material can explain our initial observation of the stronger impact of exogenous UMIs on variant detection sensitivity in cfDNA as unresolved collisions may lead to a loss of variant signal during read collapsing. Consistently, the discrepancy between the number of molecules determined using UMIs and using mapping position alone is greater in cfDNA samples when compared to FF and FFPE-derived DNA (Fig. 3D). Typical cfDNA yields in clinical setting are often no higher than 10 ng per ml of plasma [19], but the discrepancy in molecule counts is high even with such low input (i.e. ~ 9400 vs ~ 4300 molecules, 119% difference). Conversely, for FF DNA molecule count discrepancy at typical input quantities (i.e. 100–200 ng) is more limited. DNA yields from FFPE samples vary greatly and are often limited to less than 100 ng for input [20], a quantity where the molecule count difference is small.

The probability of collision also increases with the number of sequenced molecules, even if fragmentation is random (Fig. 3E). Nevertheless, at typical sequencing depths yielding read coverage below 10′000x, and for typical input amounts, collision rates did not exceed 10% for FF and FFPE DNA. Thus, we conclude that usage of UMIs for identification of individual starting molecules and read grouping provides only a limited advantage over mapping position grouping in such scenarios.

### Impact of exogenous barcoding on variant calling performance

**Fig. 4:**
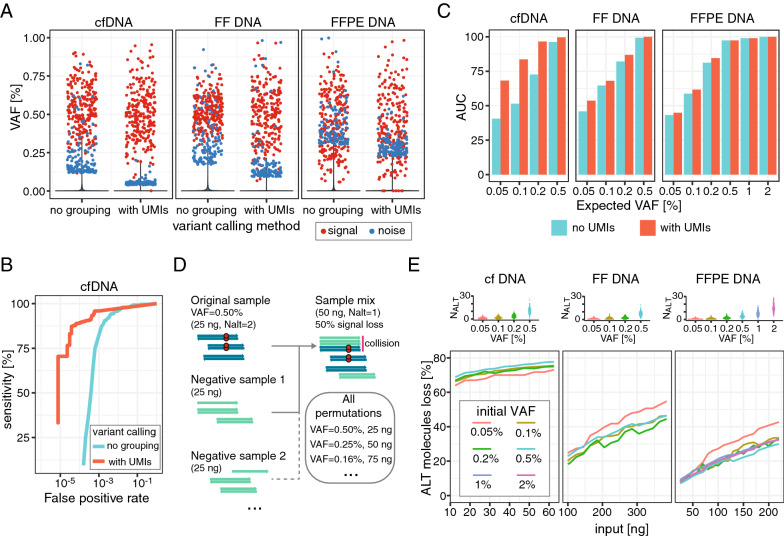
Effects of UMI usage on signal and noise. **A** Plot displaying VAF for detected true-positive (red) or false-positive (blue) variants, for variant calling without read grouping (“no grouping”) or using mapping position and UMI consensus ("with UMIs”); data is displayed for 3 types of input material (expected VAF = 0.5%). **B** ROC curve for variant calling performance in cfDNA with expected VAF = 0.2%, without read grouping or using mapping position and UMI consensus. **C** Plots displaying area under the ROC curve (AUC), using mapping positions alone (cyan) or mapping position and UMI information (red), for 3 types of input material and a range of expected VAF values. **D** Schematic representing the experimental approach for investigation of signal loss (see the “Methods” section). FASTQ files from samples with targeted variants at a given VAF (“Original sample”) were merged with FASTQ files of background DNA lacking the variants (“Negative sample”). The number of variant-supporting molecules (N_ALT_) was calculated for each dilution. An example VAF and input amounts are shown in the figure. **E** Fraction of ALT molecules lost (y axis) due to collisions as a function of input amount (x axis), for samples with different initial VAF values. Plots on top show the count of ALT molecules for each targeted variant in undiluted samples

To investigate in detail the effect of collisions on variant calling, we prepared a series of samples in which, by mixing two types of reference material, we obtained VAFs in a range of 0.05–2% (Fig. 1D). Such material was generated for different types of DNA (FF, FFPE and cfDNA) and used for library preparation, enriched for 110 loci bearing variants and sequenced. Variant calling methods utilizing UMIs allow noise suppression to levels lower than procedures not applying any read grouping, as could be observed in our dataset (Fig. 4A). However, the final noise level still depends on characteristics such as input material type, quantity, LCR, sequencing depth, among others.

As discussed, UMIs should be particularly beneficial in situations where mapping position collisions are frequent but will have little impact on performance for mapping position-based read grouping with few to no collisions. Investigating sensitivity for a fixed molecule support threshold used for variant calling, we indeed observed a strong impact of collisions for cfDNA (Fig. 1E). Similarly, we observed a reduction of the false positive rate when using mapping-position only compared to using UMIs and mapping-position, with a stronger effect for cfDNA (reduction of 0.85 × for FFPE, 0.74 × for FF and 0.45 × for cfDNA). This is expected as artifacts will be suppressed by collisions in the same manner as the real signal. Thus, in order to quantify the impact of UMIs independently of the variant calling threshold used we calculated the area under the receiver operating characteristic (ROC) curve (AUC), interpreting reduction in AUC as a measure of an overall decrease of the statistical power of the method.

As expected, when variant calling performance with and without UMI information is compared across different types of input material and variant fractions, a strong impact of UMIs is notable only for cfDNA (AUC 0.84 with UMI vs 0.52 without UMI at VAF = 0.1%), while very little effect is observed in FF and FFPE-derived samples (AUC 0.68 vs 0.64 and 0.62 vs 0.59, respectively), where collisions are less frequent (Fig. 4C). In our dataset, performance at lower VAF values, when using UMIs, was somewhat higher for cfDNA than for FF and FFPE DNA. However, this difference may stem from technical factors playing role during library preparation, such as input quality, amount and conversion rate.

The positive impact of UMIs on performance is related to improved signal retention in situations where variant information would otherwise be lost due to collisions during read deduplication. We sought to systematically estimate the extent of signal loss in various input scenarios. To this end, we diluted in silico (see “Methods” section) samples of FF DNA, FFPE DNA or cfDNA with variants present at specified VAFs (0.5–2%), to obtain a range of datasets with lower VAF values (Fig. 4D). As the diluted samples contained more total input material, the probability of collisions thus increased with decreasing VAF. We measured the number of molecules with the alternative allele of each variant (“N_ALT_”) (Fig. 4E, top). Molecule sequence was determined either by taking the UMI information into account or by relying on mapping position alone. For each condition, we then computed the fraction of variant-supporting (ALT) molecules whose signal was lost due to collisions with reference allele-bearing (REF) molecules (Fig. 4E, bottom).

As expected, overall variant signal loss was highest in cfDNA, as this is the input type with the highest collision rate, reaching up to 80% of ALT molecules lost to collisions at the maximum total input. In the clinical setting, this highlights the need for UMIs when using cfDNA to ensure variant detection sensitivity. Since the collision rate increases with input, the signal loss similarly increased with input amount for all types, confirming that collisions can pose an issue for FFPE and fresh frozen samples given use of a sufficiently large quantity of input material. Therefore, for applications such as MRD tracking, where deep sequencing of a large quantity of input material is recommended, application of molecular barcoding will have positive impact on sensitivity. Importantly, the extent of signal loss did not depend significantly on the VAF, indicating that the probability of losing variant information due to collision is similar regardless of the fraction of ALT molecules in the input within the tested range. This is expected for low VAFs (< 5%) as in these conditions the ALT molecules are much more likely to collide with a REF molecule than an ALT molecule.

Thus, UMIs may help to improve the efficiency of variant calling over mapping-position read grouping methods in datasets with frequent collisions by suppressing noise and preventing signal loss.

### Limitations of exogenous barcode usage

Our data demonstrate that the benefits of using UMIs are not universal and depend both on the method setup and the applied analytics with a range of factors affecting the performance of detection of rare alterations. Variant calling methods relying on duplex sequences require that a significant fraction of molecules have both strands converted and sequenced. Therefore, low LCR values leading to predominant conversion of only one strand per input DNA molecule will not support the level of information necessary to benefit from duplex-based noise suppression. Similar problem will occur if sequencing depth is disproportionally small relative to the number of molecules: read groups for each molecule will be too small, or will not contain both strands, preventing efficient error correction.

Design of UMI system requires taking several factors into account. First, the number of possible UMIs must be large enough to address the diversity of colliding molecules in the input sample. If the number of possible UMIs is not significantly higher than the number of colliding molecules, the molecule count may be underestimated, leading to signal loss. As collisions are less frequent for capture-based enrichment methods, much lower diversity is required for such workflows in comparison with amplicon-based assays. Conversely, some RNA-based methods might require extremely diverse UMI pools for reliable read deduplication [21]. A model for the determination of the minimum UMI length has been proposed based on the total number of DNA fragments and the distribution of allele frequencies [22]. In the case of a commonly used amplicon-based method, UMIs of length of 6–8 bp, depending on the deduplication method, were sufficient to identify all molecules when 50 ng input DNA was used [23]. Second, UMIs should have sequences distinct enough to prevent misassignment of UMI sequences due to amplification or sequencing errors (Additional file 2: Figure S1B). The minimal Hamming distance between UMI sequences should consider the overall length of the barcode. For commercially available UMIs, the estimated assignment error rates range between 0.1 and 0.4% [11, 23]. Third, the de-duplication method must allow for a certain number of mismatches to assign reads with similar, but non-identical, UMIs to the same molecule (Additional file 2: Figure S1C). Otherwise, the number of molecules will be artificially inflated due to UMI sequencing errors [11]. Finally, the size of a barcode has also an impact on the sequencing cost: 16 bp-long UMIs consume 11% of 150-bp long reads and 16% of 100 bp-long reads, reducing the effective sequencing output devoted to interrogated targets. Additionally, longer UMIs provide higher sequence degeneracy but may increase the formation of primer dimers or lead to non-specific binding events.

Certain types of errors cannot be corrected by consensus variant calling. These include symmetrical double-stranded damage to input molecules, errors occurring simultaneously on both strands at the same position, or other artifacts introduced during library preparation. An example of the latter are hairpin structures introduced during end repair and adapter ligation (Additional file 2: Figure S1D). In that case, additional palindromic or semi-palindromic sequences at read ends are present across multiple different molecules, leading to a false-positive variant call. Sequence errors occurring systematically at certain genomic positions, such as the homopolymer tracts, cannot be corrected by read grouping and deduplication prior to variant calling as they arise independently in reads derived from distinct starting molecules. Thus, UMI usage, while providing crucial advantages for variant calling in specific situations, have limitations that can be both context-specific as well as global across applications.

## Conclusions

Detecting low frequency mutations, critical for oncology applications, currently presents a significant challenge. Scarcity of input, degradation of FFPE material and high levels of noise limit the analytical performance of NGS-based methods. Improving the detection of rare mutations cannot be achieved by a single universal method across all applications. As multiple factors affect both the signal recovery and noise levels, the final choice of workflow should be guided by factors including the quantity, quality and type of input material, the library preparation method, the enrichment strategy, the sequencing technology, and the sequencing depth.

**Fig. 5:**
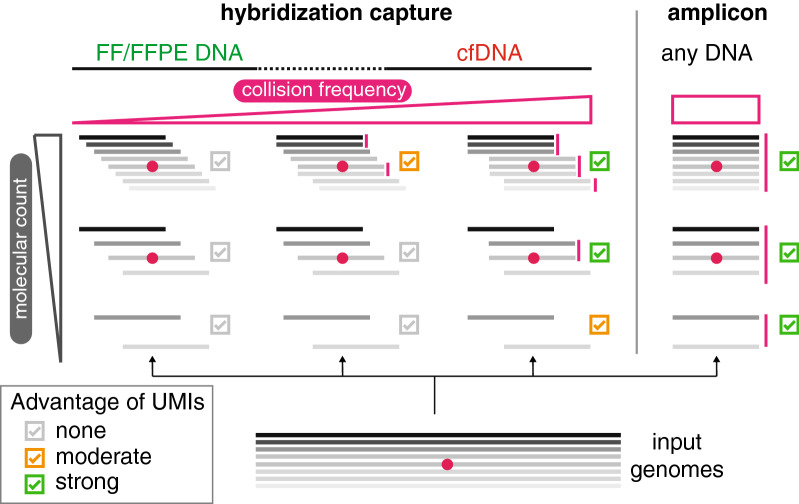
Advantage of using UMIs in various experimental setups. Schematic summary of experimental setups where UMIs provide advantage in performance over the mapping position-based grouping; for the same set of input genomes, benefit of using UMIs will depend on the collision frequency (determined by sequenced fragment end distribution) and the molecular count (determined by input amount, LCR and sequencing depth)

UMIs are a common addition to sequencing workflows intended to improve detection of low frequency variants. Using systematic calculations, we demonstrate specific, clinically-relevant scenarios in which the usage of UMIs is indeed beneficial for improved variant calling performance (Fig. 5), particularly when a significant collision rate is observed in the sequencing data. Since in many experimental setups the collision rate is not a major factor limiting sensitivity, UMI usage should be carefully considered on a case-by-case basis rather than adopted as default. We believe that our empirical demonstrations of UMI utility will help in guidance of the UMI system choice for various NGS applications.

## Supplementary Information

**Additional file 1.** Supplementary Table 1.

**Additional file 2.** Figure S1.

## Data Availability

Sequencing data is available in NCBI Sequence Read Archive (https://www.ncbi.nlm.nih.gov/sra) under submission number PRJNA937211.
