# Supplementary material for: Benefits of applying molecular barcoding systems are not uniform across different genomic applications
Source: J Transl Med. 2023 May 5;21:305. doi: 10.1186/s12967-023-04160-0 (PMC10163729; doi:10.1186/s12967-023-04160-0)
Supplement: Supplementary file 2 — Additional file 2. Figure S1. [file 12967_2023_4160_MOESM2_ESM.docx]

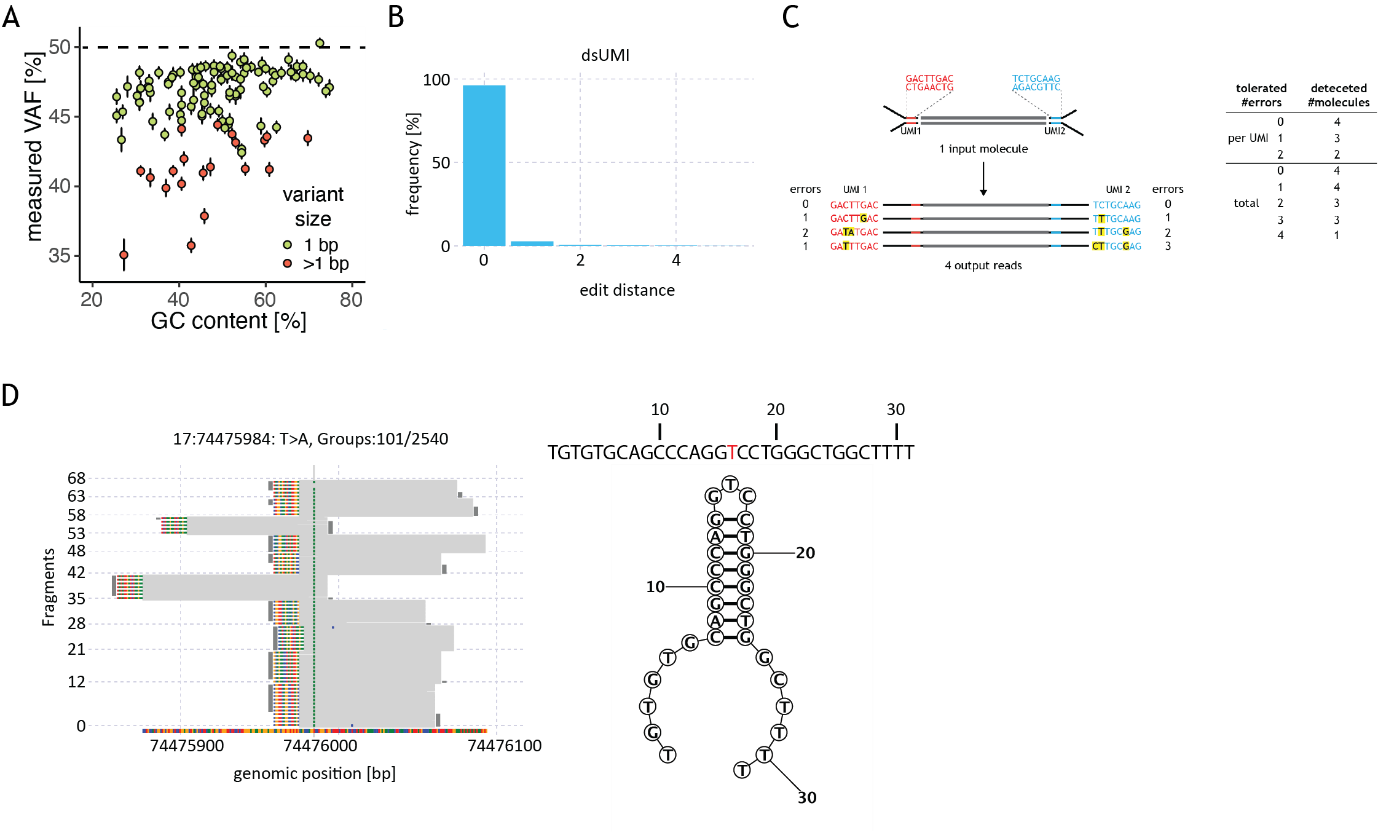
**Additional Figure 1**

**Figure S1. (A)** Signal (VAF, y axis) measured using hybridization capture assay, for 110 heterozygous variants present in the context of varying GC content and variant size; deviation from expected VAF is stronger for variants that are larger and in GC-poor regions. **(B)** Observed frequencies of edit distances between UMI sequences belonging to the same molecular group. A small fraction of UMIs show an edit distance of 1-4 due to sequencing errors in the UMI sequence. **(C)** Impact of allowed UMI mismatches to assign read pairs to the same group on the number of detected molecules; mismatches can be allowed per a single UMI or per UMI pair (“total”). **(D)** Example of a false positive call due to a palindromic genomic sequence leading to hairpin formation in the library. The left panel shows 10 of the groups supporting the T to A mutation in green (a total of 101 groups are supporting the variant, out of 2540 groups covering this position). Light gray lines represent fragments, the sequences on the left of the groups are the UMI sequences and the vertical dark gray lines indicate if the fragments originate from the plus or minus strand of the DNA molecule. The signal shows consistently in groups, even when both strands are present. However, the variant is found at a non-random position within the fragments. The right panel shows the predicted structure by RNAstructure^16^, showing a strong hairpin with the mutated T in the unpaired part of the loop (red).
